# Growth rate and rupture rate of unruptured intracranial aneurysms: a population approach

**DOI:** 10.1186/1475-925X-8-11

**Published:** 2009-06-18

**Authors:** Liang-Der Jou, Michel E Mawad

**Affiliations:** 1 Department of Radiology, Baylor College of Medicine, Houston, Texas, USA

## Abstract

**Background:**

Understanding aneurysm growth rate allows us to predict not only the current rupture risk, but also accumulated rupture risk in the future. However, determining growth rate of unruptured intracranial aneurysms often requires follow-up of patients for a long period of time so that significant growth can be observed and measured. We investigate a relationship between growth rate and rupture rate and develop a theoretical model that can predict average behavior of unruptured intracranial aneurysms based on existing clinical data.

**Methods:**

A mathematical model is developed that links growth rate and rupture rate. This model assumes a stable aneurysm size distribution so the number of aneurysm ruptures is balanced by the growth of aneurysms. Annual growth rates and growth profiles are calculated from a hypothetical size distribution and data from a previous clinical study.

**Results:**

Our model predicts a growth rate of 0.34–1.63 mm/yr for three different growth models when the rupture rate at 10 mm is 1%. The growth rate is 0.56–0.65 mm/yr if annual rupture rate averaged over all aneurysm sizes is assumed to be 2%. The peak of aneurysm size distribution coincides with a period of slow growth between 5 mm and 8 mm.

**Conclusion:**

This mathematical model can be used to predict aneurysm growth rate, and the results are consistent with previous clinical studies. Predictions from both hypothetical and clinical cases agree very well. This model explains why some aneurysms may grow into a stable size and remain so without rupture.

## Background

Rupture of an intracranial aneurysm is devastating; however, prevention remains a challenge since most aneurysms are asymptomatic before rupture. Rupture risk is difficult to assess even if an aneurysm is detected. Each aneurysm behaves differently, so the only way to study this cerebrovascular disease is through its natural history. Natural history of intracranial aneurysms can help us to understand the course of aneurysm development and allow proper assessment of aneurysm rupture risk. Ultimately, patients can benefit from rational decisions regarding their treatments based on their individual risk.

A large clinical study on unruptured intracranial aneurysms, the International Study of Unruptured Intracranial Aneurysms (ISUIA), has studied more than 4000 aneurysm patients and raised serious interests on risk factors and rupture rate of unruptured aneurysms [1,2]. They found that rupture rate increased with aneurysm size in both retrospective and prospective studies, and rupture rate was higher for patients with a history of subarachnoid hemorrhage. Rupture rate assesses immediate rupture risk, and growth rate allows prediction of accumulated rupture risk and evaluation of treatment timing. While large studies of this kind have provided critical information on rupture rate and risk factors, few studies have analyzed aneurysm growth rate in the same rigorous manner. Most studies analyzed angiographical images of aneurysms retrospectively and had very short follow-up periods [3,4]. Ethical concerns and small growth rates are among the major reasons that prohibit a large prospective clinical study on growth rate. It often requires a few years for aneurysms to grow at least 1mm larger so that growth can be appreciated on angiograms[5]. Intra- and inter-observers variation may also seriously affect the accuracy of growth rate [6,7].

Koffijberg et al. found that intracranial aneurysms did not grow constantly and prediction of their behavior might be extremely difficult [8]. Markov chain models have been used to study the natural history of unruptured intracranial aneurysms and benefit of prophylactic treatment [9]. These reports all relied on estimated aneurysm initiation rate, growth rate, and rupture rate [9,10].

Rupture rate and growth rate are two seemingly unrelated concepts. Will it be possible to determine the growth rate of unruptured intracranial aneurysms from rupture rates in previous clinical studies? In this report, we explore and investigate the possible mathematical relationship between rupture rate and growth rate. We employ a different approach and intend to derive aneurysm growth rate from clinical data. A simple mathematical model with only a few parameters can give us a reasonable description of average aneurysm behavior without organizing large clinical studies to understand the natural history of unruptured intracranial aneurysms.

## Methods

**Figure 1:**
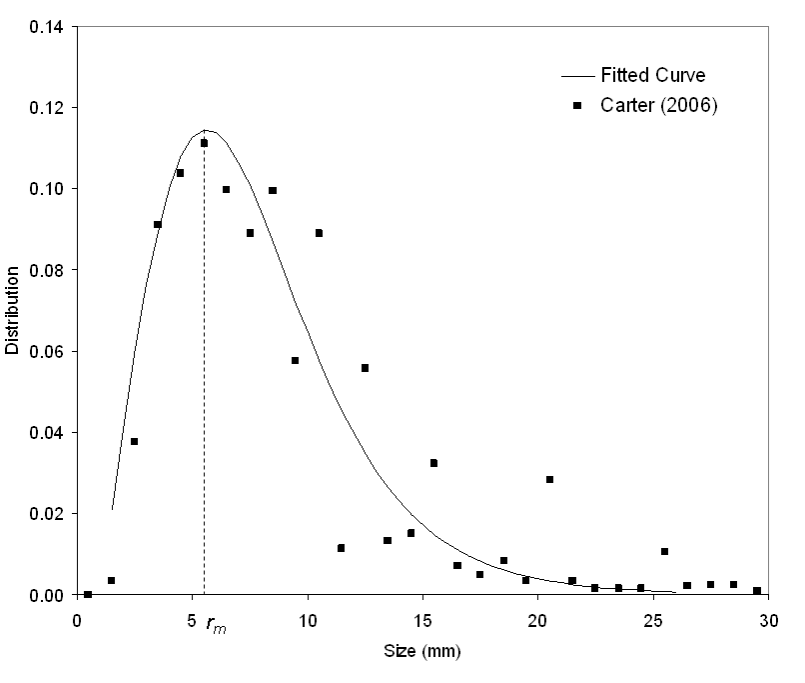
**Size distribution of unruptured aneurysms**. The mode of *N(r) *is at *r*_*m*_.

We first assume that there is a stable size distribution of unruptured intracranial aneurysms, *N*, and this distribution is characterized by aneurysm size, *r*. This aneurysm size *r *is the maximum aneurysm dimension used clinically so the size distribution from existing literature can be used. We choose to ignore individual risk factor at this time and study the entire aneurysm patient population as a whole. Therefore, this distribution may represent the entire aneurysm patient population in the world, or in a country where the population is large enough that the size distribution does not change significantly over time. A representative distribution is provided in Figure 1, and the mode of this distribution is denoted as *r*_*m*_. Size distribution is essentially a histogram of aneurysm size. All unruptured aneurysms are characterized by their sizes at 1 mm interval. Data from Carter et al. [11] are shown in Figure 1 along with a fitted curve. The exact mathematical formula for the curve will be discussed later.

Suppose that the rates of growth and rupture are *g *and *f*, respectively, and both of them are functions of aneurysm size only. Specifically, grow rate *g(r) *is the percentage of aneurysms growing into a size *r *+ Δ*r *from size *r*, for example, from 10 mm to 11 mm. If the total number of these patients is *C*, the number of patients with an aneurysm of size *r *will be *C N(r)*. The number of aneurysms that rupture in a time period Δ*t *is *C N(r) f(r)Δt*, and the number of aneurysms that grow to a larger size *r + Δr *during the same time period is *C N(r) g(r) Δt*. Therefore, one can arrive at a mathematical equation for the aneurysms:

(1)

where *t *is the time. The first term on the left hand side describes the increase of the number of aneurysms with time, and the second term on the left hand side is the net change of the number of aneurysms due to growth. A number of aneurysms will grow into a bigger size (>*r*) and a number of smaller aneurysms (<*r*) will grow to the size *r*, and the net result will be their difference, . Net change of these aneurysms is balanced by the number of aneurysm ruptures on the right hand side. Equation (1) essentially describes the conservation of unruptured aneurysms at the size *r*. Once an aneurysm bleeds, it will become a ruptured aneurysm, and its behavior is different from unruptured aneurysms. Ruptured aneurysms need to be dealt with differently since it involves not only the aneurysms that rupture now, but also the aneurysms that ruptured in the past; patients suffered from ruptured aneurysms may or may not survive. If we consider only the cases that rupture within a short time period Δ*t*, then the distribution of ruptured aneurysms is , and the average annual rupture rate is . *h *will be used for this average annual rupture rate over al aneurysm sizes, and *h *is actually a weighted average of rupture rate *f(r)*.

Because we have assumed that unruptured aneurysms have a stable size distribution, *N *is not a function of time; the total number of aneurysms, *C*, may still change with the time because of formation of new aneurysms or loss of aneurysms due to ruptures. Equation (1) can be rewritten as



or

(2)

One can easily calculate the growth rate *g(r) *if the aneurysm distribution, *N(r)*, aneurysm rupture rate, *f(r)*, and increase of total number of aneurysms, , are known. Because *N(r) *is an aneurysm size distribution, integration of *N(r) *over all aneurysm sizes will be the unity, that is, 

Thus, integration of Equation (2) over the entire aneurysm range yields

(3)

Equation (3) shows that initiation of new aneurysms *CN(r = 0) g(r = 0) *is tied to the average rupture rate (*h*) and temporal change of patient population (). Therefore, one cannot assume both the growth rate and rupture rate at the same time, or Equation (3) will be invalid. Initiation and growth of aneurysms are important in maintaining a stable aneurysm size distribution. The change of *C *also reflects the deaths of these patients from other diseases or life events.

Although *g(r) *is the percentage of aneurysms that grow, it is related to aneurysm size growth. Considering that the aneurysms at the size *r *is *N(r)*, the average size of these aneurysms after a time period Δ*t *is . *CN(1-gΔt) *is the number of aneurysms remaining at the size *r*, and *CNgΔt *is the number of aneurysms growing into the size *r *+ Δ*r*. So the change of average size for these aneurysms is *g*(*r*)Δ*tΔr*, which describes the increase of mean aneurysm size within a given time period. For example, there are 100 aneurysms in the 5 mm range, and the mean aneurysm size for these aneurysms is 5 mm. 20% of these aneurysms grow into the 6 mm range, then the mean aneurysm size for these 100 aneurysms is 5.2 mm (=(80*5+20*6)/100). Thus, 20% of aneurysms that grow lead to a growth rate of 0.2 mm/year. This argument works only when the time period (Δ*t*) is small enough that no aneurysms grow into the 7 mm range. The average time needed for the aneurysms size *r *to grow Δ*r *is  and the time that it takes an aneurysm to grow from *r *= 0 to *r *= R is 

These equations establish a mathematical model for our analysis. In the following, we will analyze average aneurysm growth behavior based on these relationships.

## Results

Our model basically provides a link between growth rate and rupture rate; however, the aneurysm size distribution is a pre-requisite to the calculation of growth rate. Thus, we assume a profile for the aneurysm size distribution: *N*(*r*) = *ar*/(1+*kr*^2^)^2^. This is a fitted curve of the data in [11]. In the profile, *k *controls the peak of the profile, *r*_*m*_, and *a *is determined by  An example for *r*_*m *_= 5.77 is shown in Figure 1. There is only one parameter in the size distribution so determining the size distribution is easy if the mode (*r*_*m*_) can be found from clinical data. Nonetheless, the exact mathematical formula is not used in the simulations, and only the numerical values at each size group are used.

In the following, we discuss three different cases for which we derive the growth rates from rupture rates. In these cases, the rupture rate *f *is proportional to various power of aneurysm size.

### A) Rupture rate proportional to aneurysm size

**Figure 2:**
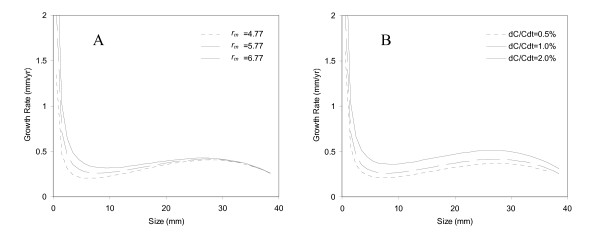
**Growth rates for (A) different *r*_*m *_and (B) different aneurysm population growth *dC/Cdt *for Case A**.

We first assume that rupture rate is proportional to aneurysm size, so *f*(*r*) = *αr*, where *r *is the aneurysm size. *α *is a constant and is adjusted to achieve a certain rupture rate at a specific aneurysm size. Initially, we assume a 1% annual rupture rate for 10 mm aneurysms [12]. We can adjust *α *so that the rupture rate is 1% for aneurysms of 10 mm in size. One can arrive at the growth rate by integrating Equation (2) numerically, and the results for different *k *values are shown in Figure 2. Figure 2A shows how unruptured aneurysms grow on average. It takes approximately 30 years for an aneurysm to grow 10 mm. There is a local minimum growth rate, and this local minimum growth rate is at 6.5 mm for *r*_*m *_= 4.77 mm, 7.5 mm for *r*_*m *_= 5.77 mm, and 9 mm for *r*_*m *_= 6.77 mm. Also, this local minimum growth rate is between 0.2 – 0.3 mm/yr and increases with *r*_*m*_. In other words, only 20–30% of aneurysms at this size will grow 1 mm larger in a year. Note that there is also a local maximum growth at *r = 28 mm*, and the growth rate declines for aneurysms greater than 28 mm. Decline of growth rate beyond 28 mm may be explained as a limitation on the production of new collagen for growth. Still, these giant aneurysms rupture more often than smaller aneurysms, and their rupture risks are not reduced simply because of smaller growth rates.

Because growth rate depends on the change of total number of aneurysms (*dC/dt*), a sensitivity analysis is conducted for different patient population growth rate (0.5%, 1%, and 2%). The patient population growth, *dC/Cdt*, is different from aneurysm growth rate. Aneurysm growth rate describes how fast an aneurysm grows into a larger size, and the patient population growth reflects the change of total number of aneurysm patients. Suppose that the aneurysm prevalence is 2% in the U.S., then aneurysm patient population growth rate will be 1%, which is the same as the general population growth rate in the U.S. Aneurysm patient population growth needs to be the same as the general population growth so the prevalence of aneurysms is a constant.

The sensitivity of aneurysm growth rate to patient population growth rate is shown in Figure 2B. The growth rate is 0.32 mm/yr, 0.37 mm/yr, 0.47 mm/yr at 20 mm for the patient population growth rates of 0.5%, 1%, and 2%, respectively. Patient population growth rate of 1% is used in all the following analyses unless stated otherwise.

### B) Rupture rate proportional to the square of aneurysm size

In this case, rupture rate is proportional to the square of aneurysm size, so *f*(*r*) = *αr*^2^. *α *is adjusted so that annual rupture rate is 1% for 10 mm aneurysms. For spherical aneurysms, this rupture rate is proportional to aneurysm surface area.

### (C) Rupture rate proportional to the cube of aneurysm size

A previous study hypothesized that the rupture rate of an aneurysm is proportional to the aneurysm volume [13]. They argued that vessel thickness is proportional to vessel size and weakest sites of an aneurysm are proportional to the total cell mass (thickness × surface area), so rupture rate is proportional to the aneurysm volume. In this case, rupture rate is assumed to be proportional to the cube of aneurysm size: *f*(*r*) = *αr*^3^. *α *is adjusted so that rupture rate is 1% for 10 mm aneurysms. For spherical aneurysms, this rupture rate is proportional to aneurysm volume. Aneurysm growth in this case is obviously faster than previous two cases.

**Figure 3:**
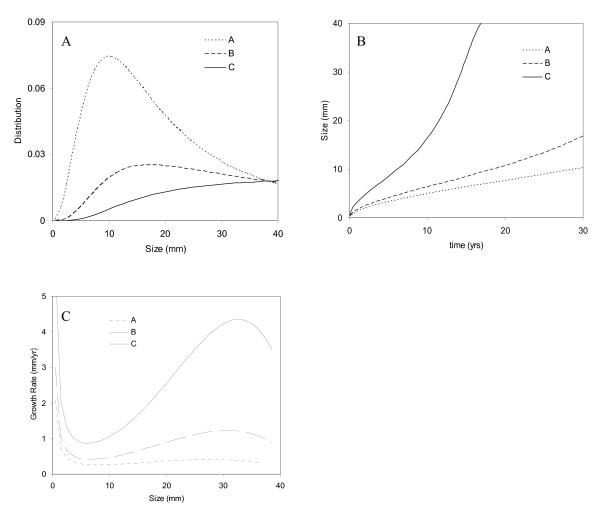
**(A) Ruptured aneurysm size distribution, (B) growth curve, and (C) growth rates for three different cases**. Rupture rate is proportional to 1^st^, 2^nd^, and 3^rd ^order of aneurysm size for Case A, B, and C, respectively. *r*_*m *_= 5.77.

Figure 3 shows the ruptured aneurysm distribution, growth curve, growth rate for all three cases (A-C). In Figure 3A, there is a size at which more aneurysms rupture for Case A and B, but it is not obvious for Case C. The number of ruptured aneurysms declines very slowly when *r *is greater than *18 mm *for Case B.

Growth curves in Figure 3B present the time it takes for an aneurysm to grow to a certain size. The analysis shows that it needs 20 and 9 years for an aneurysm to grow to 10 mm for Case B and C, respectively. There are local minimum and maximum growth rates for aneurysms from Figure 3C. For Case C, the growth rate varies from 0.5 mm/yr at 5 mm to 4 mm/yr at 30 mm.

**Figure 4:**
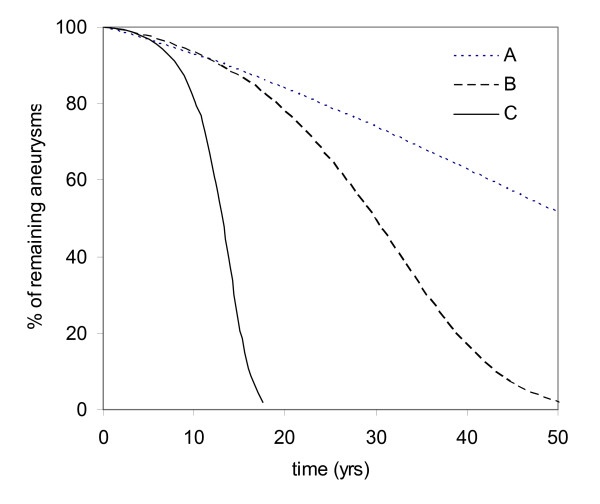
**Survival rate for 8 mm aneurysms for Cases (A-C)**.

Figure 4 compares the survival rate (or non-rupture rate) of 8 mm aneurysms under these three cases (A-C). More than 50% of aneurysms do not rupture for Case A after 50 years, 50% for Case B at 30 years, and less than 30% for Case C at 15 years. The annual rupture rates for these cases are 1%/yr for the case A, 2%/yr for Case B, and 5.5%/yr for Case C. The increase of rupture rate from 1% to 5.5% is mostly due to a rapid increase of aneurysm size in Case C.

**Figure 5:**
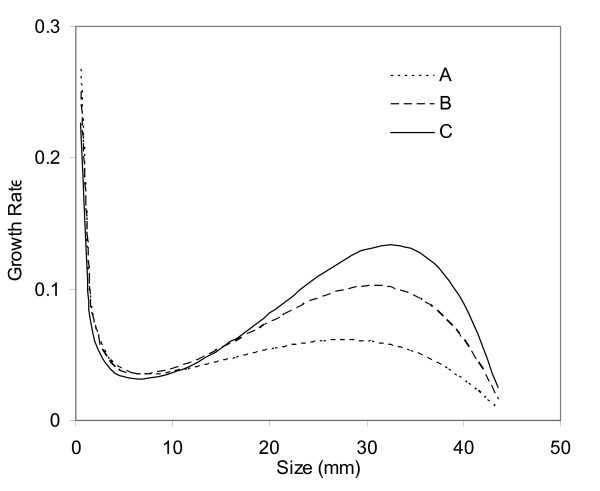
**Growth rate for an average of 2% rupture rate (*h *= 2%/yr)**.

Because the long-term survival rate (averaged over a long period) is easier to obtain from clinical reports than the short term rupture rate, *f(r)*, analyses are done at the long term annual rupture rate of 2%/year for all cases (*h *= 2%/yr) [14]. These results are shown in Figure 5. Case C has twice the growth rate of Case A at 30 mm. The growth behaviors for aneurysms smaller than 10 mm are very similar in all three cases, so it will be difficult to determine growth behavior from studies of aneurysms smaller than 10 mm. This can have a great implication on design of clinical studies. One is unlikely to learn aneurysm behavior from aneurysms smaller than 10 mm in a long-term study because the differences between these cases are so small.

A comparison of these cases is shown in Tables 1 and 2. In the tables, two different rupture rates are used: one assuming a rupture rate of 1% for 10 mm aneurysms (*f*_10 *mm *_= 1%/yr) and the other having a 2% annual rupture rate averaged over all aneurysm sizes (*h *= 2%/yr). Our model predicts a growth rate of 0.34–1.63 mm/yr for unruptured aneurysms with *f*_10 *mm *_= 1%/yr and a growth rate of 0.56–0.65 mm/yr for *h *= 2%/yr.

**Table 1:** Differences in the mean growth rate (mm/yr) for three different cases.

	Case A	Case B	Case C
*f*_*10 mm *_= 1%/yr	0.34	0.65	1.63
*h *= 2%/yr	0.56	0.62	0.52

**Table 2:** Differences in the time (yrs) for an aneurysm to grow to 10 mm after initiation.

	Case A	Case B	Case C
*f*_*10 mm *_= 1%/yr	28.9	18.4	8.9
*h *= 2%/yr	20.4	19.4	19.1

Significant differences can be seen between Cases A and C.

**Figure 6:**
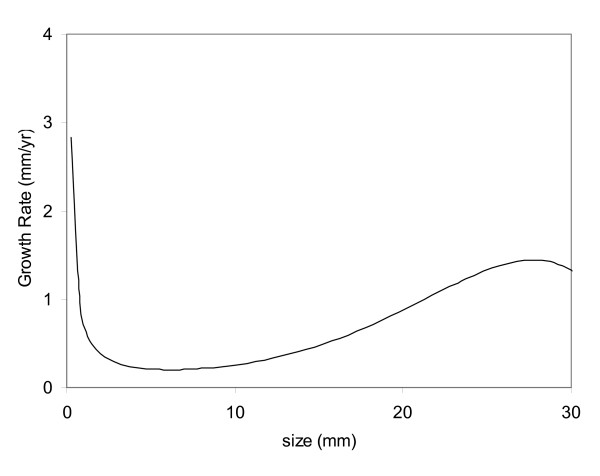
**Calculated growth rate using ruptured and unruptured aneurysm size distribution from Weir et al**. A long period of slow growth exists for aneurysms smaller than 10 mm.

We apply our mathematical model to calculate the growth rates using the size distribution of unruptured and ruptured aneurysms in Weir et al. [15]. Results are presented in Figure 6. In their report, the peak of the size distributions (*r*_*m*_) is 4.6 mm and 8.3 mm for unruptured and ruptured aneurysms, respectively. Our results show a 0.61 mm/yr growth rate for *h *= 2%/yr and 0.37 mm/yr for *h *= 1%/yr.

## Discussion

Obtaining size distribution of unruptured aneurysms needs at least one angiographical examination for a large number of aneurysm patients [15]. Determining rupture rate requires additional follow-up on these patients either by interviews or questionnaires for their current conditions [1,2]. Even then, the results represent only the average rupture rate of a specific range of initial aneurysm size, assuming that there is no significant growth during the study period. At least two angiographical examinations are required to determine the growth rate for each patient [3-5]. Since only a small number of aneurysms actually grow, the effort leading to determination of aneurysm growth rate is enormous. We have derived a model that describes the relationship between growth rate and rupture rate of unruptured intracranial aneurysms. Based on the assumption that there is a stable aneurysm size distribution, we learn that one cannot assume both growth rate and rupture rate in an analysis, or the model is invalid.

Our model does not deal with any specific aneurysm or aneurysms at a particular location, but it predicts average behavior of entire aneurysm population. Based on our analysis, aneurysm size is no longer the only consideration for rupture risk. For Case A, an 8 mm aneurysm growing at 0.22 mm/yr behaves just like any other 8 mm aneurysms. However, a 5 mm aneurysm with a growth rate of 0.3 mm/yr is growing 50% faster than average 5 mm aneurysms, and this increase in growth rate may out-weight aneurysm size in assessment of rupture risk. Therefore, the 8 mm aneurysm may have a smaller rupture risk than a 5 mm aneurysm in this case; it becomes important when both aneurysms are found in the same individual and one of them needs to be treated immediately.

Clinical experiences show that intracranial aneurysms either grow to rupture after development or grow into a certain size without rupture. Our model explains why many aneurysms grow into a certain size and remain stable without rupture for a long period of time. Initial aneurysm development is often followed by rapid growth at early stage, and then the growth is slowed down between 5 and 8 mm, as depicted in Figure 6. This slow growth period coincides with the peak of unruptured aneurysm size distribution in Figure 1, implying that only a very small percentage of aneurysms sized between 5–8 mm are growing. Thus, more aneurysms are found at this size range and their growth is slow. Aneurysm growth regains its pace when aneurysm size is greater than 10 mm, and growth accelerates until it is larger than 30 mm. Fast growth and increasing rupture rate render fewer aneurysms at larger sizes. Note that the end of this slow growth period between aneurysm size of 5–8 mm happens to be near the critical aneurysm size [16], and this might also indicate a change of aneurysm growth behavior and beginning of an increasing rupture risk. Nevertheless, our model shows the same behavior both for a hypothetical model and from clinical data [15].

Growth rate of unruptured aneurysms can be related to rupture rate. Therefore, we can calculate growth rate given a specific rupture rate. Our model gives a growth rate of 0.34–1.63 mm/yr when *f*_*10 mm *_= 1%/yr. However, our model shows a narrow range of growth rate (0.56–0.65 mm/yr) for *h *= 2%/yr. By assuming a 2% annual rupture rate averaged over entire aneurysm size range, the difference among three cases is smaller for small aneurysms, and their difference would be difficult to quantify based on clinical data. Juvela et al. reported a 0.31 mm/yr growth rate in 45% of patients with a mean follow-up of 19 years [5]; however, nearly 90% of patients in their study had subarachnoid hemorrhage at other locations before the study began. Phan et al. reported that 7% of unruptured intracranial aneurysms grew during an average follow-up period of 50-month, and all growing aneurysms were greater than 9 mm [3]. We learn from Figure 6 that unruptured aneurysms smaller than 9 mm grow 0.18 mm/yr (or 0.75 mm in 50 months), and few aneurysms can grow 1 mm larger within 50 months, consistent with Phan's finding. Therefore, observation of growth of small aneurysms is infrequent [17], and only a long-term study can reveal significant growth [5].

Because of the connection between rupture rate and growth rate, high rupture rate can only be explained by faster aneurysm growth. Among the cases that we have studied, Case C, in which rupture rate is proportional to the cube of aneurysm size, gives the fastest growth. However, it generates an unusual ruptured aneurysm size distribution in Figure 3A. Mean ruptured aneurysm size is larger than 20 mm, and this clearly differs from previous data. Aneurysm growth of 10mm^3^/yr suggested in another study is, however, too restrictive since it yields little growth for large and giant aneurysms [10]. Therefore, rupture rate probably will not increase as fast as in Case C.

Rupture risk involves a short-term rupture rate and an increase of rupture rate associated with aneurysm growth. Case B gives a 75% survival rate at 23 years, similar to 70% survival rate at 25 years reported by Juvela et al. [12]. However, 84% of aneurysms survive without rupture after 25 years when the growth is not considered, and rupture risk is actually underestimated by 35%. Therefore, the rupture rate derived from clinical survival rate may need to be lowered to reflect the growth of aneurysms. Since the surface area represents the amount of tissue in an aneurysm, the assumption that rupture rate is proportional to the square of aneurysm size (Case B) seems to be logical as long as wall thinning is not significant.

Our theoretical model has several advantages. First, the model parameters can be determined from clinical data. Unruptured aneurysm distribution is available from many studies [1,2,11,15,18]. The parameter *α *can be computed from average rupture rate. The resulting model then predicts average behavior of unruptured aneurysms at any given size. Second, we can compute the growth rate from rupture rate easily. Growth of unruptured aneurysms is very difficult to study. Very few aneurysms grow, and they grow very slowly when they do grow. Therefore, our understanding of aneurysm growth often relies on a small number of cases [3-5]. Our model does help us in understanding general growth behavior of unruptured aneurysms.

Limitations exist in our model. We focus on gross behavior of unruptured aneurysms and ignore individual risk factors that might ultimately dictate rupture. The growth rate *g *and rupture rate *f *are averaged over all risk factors, so our model can be used to predict only the average behavior of unruptured aneurysms. The biological ages of these aneurysms or patients are not explicitly represented in the model. However, inclusion of biological age of an aneurysm is possible [8], but the final model may be so complex that model parameters cannot be obtained or verified from existing data. Since patient age is considered in every aspect of a clinical decision, the benefit from inclusion of patient age explicitly may be minimal.

## Conclusion

We have formulated a theory by which growth rate of unruptured intracranial aneurysms can be determined from aneurysm size distribution and rupture rate. This mathematical model can be used to predict aneurysm growth rate, and the results are consistent with previous clinical studies. Aneurysm behaves similarly under hypothetical conditions and from clinical date. This model also explains why some aneurysms may grow into a certain size and remain so without rupture. Rupture risk may also be underestimated if aneurysm growth is not accounted for.

## Competing interests

The authors declare that they have no competing interests.

## Authors' contributions

MEM and LDJ together developed the idea that growth rate and rupture rate are two related concepts. LDJ derived the mathematical model for analyses and drafted the manuscript. MEM reviewed clinical literature and revised the manuscript. All authors have read and approved the final manuscript.
